# Antagonistic properties of *Lactiplantibacillus plantarum* MYSVB1 against *Alternaria alternata*: a putative probiotic strain isolated from the banyan tree fruit

**DOI:** 10.3389/fmicb.2024.1322758

**Published:** 2024-02-09

**Authors:** R. Vasundaradevi, M. Sarvajith, Rakesh Somashekaraiah, Adithi Gunduraj, M. Y. Sreenivasa

**Affiliations:** Applied Mycology Laboratory, Department of Studies in Microbiology, University of Mysore, Mysuru, India

**Keywords:** tropical fruits, phytopathogen, *Alternaria* conidia, antifungal activity, cell-free supernatant, organic acids, succinic acid

## Abstract

*Alternaria alternata*, a notorious phytopathogenic fungus, has been documented to infect several plant species, leading to the loss of agricultural commodities and resulting in significant economic losses. Lactic acid bacteria (LAB) hold immense promise as biocontrol candidates. However, the potential of LABs derived from fruits remains largely unexplored. In this study, several LABs were isolated from tropical fruit and assessed for their probiotic and antifungal properties. A total of fifty-five LABs were successfully isolated from seven distinct fruits. Among these, seven isolates showed inhibition to growth of *A. alternata*. Two strains, isolated from fruits: *Ficus benghalensis*, and *Tinospora cordifolia* exhibited promising antifungal properties against *A. alternata*. Molecular identification confirmed their identities as *Lactiplantibacillus plantarum* MYSVB1 and MYSVA7, respectively. Both strains showed adaptability to a wide temperature range (10–45°C), and salt concentrations (up to 7%), with optimal growth around 37 °C and high survival rates under simulated gastrointestinal conditions. Among these two strains, *Lpb. plantarum* MYSVB1 demonstrated significant inhibition (*p* < 0.01) of the growth of *A. alternata*. The inhibitory effects of cell-free supernatant (CFS) were strong, with 5% crude CFS sufficient to reduce fungal growth by >70% and complete inhibition by 10% CFS. Moreover, the CFS was inhibitory for both mycelial growth and conidial germination. CFS retained its activity even after long cold storage. The chromatographic analysis identified organic acids in CFS, with succinic acid as the predominant constituent, with lactic acid, and malic acid in descending order. LAB strains isolated from tropical fruits showed promising probiotic and antifungal properties, making them potential candidates for various applications in food and agriculture.

## 1 Introduction

*Alternaria*, a widespread fungus encompassing both pathogenic and saprophytic species, poses a substantial threat to agriculture and the economy. It causes an extensive loss and spoilage of postharvest fruits, vegetables, and cereals (Gabriel et al., 2017). There are more than 60 known species of *Alternaria*, of which *A. alternata* comprises seven distinct pathotypes, each producing host-specific toxins, that result in severe diseases in different host plants (Gai et al., 2023). *A. alternata* frequently contaminates agricultural produce such as tomatoes, potatoes, melons, cucumbers, citrus, and apples (Stewart et al., 2013; Tanahashi et al., 2016; Armitage et al., 2020). Notably, *A. alternata* colonization primarily exploits the pre-existing surface lesions, caused by bruising, or ripe-fruit cracking and eventually causes diseases like Alternaria black rot, leaf spot, and brown spot (Masunaka et al., 2005; Armitage et al., 2020). Currently, chemical agents are recommended to manage or prevent *Alternaria* infection (Steglińska et al., 2022). However, most of the chemicals carry risks of toxicity to both humans and the environment. It can deplete the local microbial community and develop resistance in pathogens (Steglińska et al., 2022). To mitigate these impacts, there is a growing need for environmentally friendly alternatives to chemical treatments.

Lactic acid bacteria (LAB) represent a promising option for biocontrol applications. They are commonly encountered in a wide array of sources such as dairy products, meat, vegetables, fruits, and wine (Somashekaraiah et al., 2019, 2021; Kumari et al., 2022). The widespread distribution of LAB suggests a greater adaptability to diverse environments and versatile metabolic pathways (Garcia et al., 2016). Notably, LAB holds the status of both Generally Recognized as Safe (GRAS) and Qualified Presumption of Safety (QPS), which accentuates their significance in the probiotics and biological control agents (Adithi et al., 2022). LABs exhibit a remarkable ability to produce multiple antimicrobial compounds, offering an environmentally friendly and effective means of countering specific microbial threats, thereby playing a pivotal role in developing biocontrol capabilities. Certain LAB strains, known for their antifungal properties, have also demonstrated as natural preservatives in food applications. For instance, LAB isolated from kimchi showed greater inhibition to growth of *Cladosporium* sp. YS1, *Penicillium crustosum* YS2, and *Neurospora* sp. YS3 than calcium propionate. While *Pediococcus pentosaceus* and *Lactobacillus plantarum* isolated from Tunisian grapes showed greater detoxification capability to ochratoxin A produced by *Aspergillus niger aggrégats* and *Aspergillus carbonarius* (Taroub et al., 2019).

Fruits, among all sources of probiotics, are naturally safe and necessitate minimal processing (Garcia et al., 2016; Rodríguez et al., 2021). The distinctive chemical composition of fruits provides a unique microbial niche, fostering the isolation of a wide variety of LABs with diverse metabolic functions, including antifungal properties. Raw fruits and their byproducts exhibit inherent similarities with the human gastrointestinal tract, particularly, acidity, and the presence of antinutritional factors such as tannins and phenols (Vitali et al., 2012). It is important to note that tropical fruits have the potential to be a natural source of bioactive compounds with antimicrobial properties (Maria, do Socorro et al., 2010). Furthermore, these fruits are naturally adapted to their intrinsic characteristics, which could potentially enhance their survival during processing and storage (Garcia et al., 2016). Despite this, most of the studies involving LAB are derived from dairy or fermented food products. There is still lack of investigations on antifungal properties of LAB, sourced from fruits (Garcia et al., 2016). Given that *A. alternata* is a cosmopolitan, endophytic fungus primarily found in soil, we sought to explore tropical fruits as a source for selecting LAB for antifungal properties against *A. alternata.* By exploring these fruits, it is possible to reduce dependency on synthetic antimicrobials and promote natural alternatives. Moreover, the probiotic property of LAB strains offers an added advantage for its safe consumption by humans.

Accordingly, the objectives of this study were designed to (i) isolate LAB from previously unexplored tropical fruits, (ii) evaluate the isolated LAB for *in vitro* probiotic properties, (iii) screen and evaluate antifungal activity against *A. alternata*, and (iv) discern and identify the specific antifungal component produced by LAB. The isolation process targeted seven distinct tropical fruits as potential sources of LAB. The selected LAB was studied for growth kinetics, tolerance to bile, salt, and phenol, aggregation ability, and a comprehensive safety evaluation including antibiotic resistance and hemolytic activity. Following a rigorous screening and meticulous selection process, the most potent LAB strain was studied *in vitro* to evaluate its antifungal activity against *A. alternata*.

## 2 Materials and methods

### 2.1 Isolation and characterization of LAB

#### 2.1.1 Isolation of LAB from tropical fruits

The LAB was isolated from locally grown fruits in Karnataka, India specifically in the Mysuru and Mandya districts. Most of the fruits were picked during the Spring of 2020 for isolation. The fruits were selected primarily based on local availability, and rarity, emphasizing the exploration of previously unexplored varieties. The selected fruits included: *Musa* sp. cv. Nanjangud rasa bale (Nanjangud rasabale), *Annona muricata* (soursop), *Ficus glomerata* (cluster fig), *Couroupita guianensis* (cannonball tree), *Solanum nigrum* (black nightshade), *Ficus benghalensis* (Banyan tree), *Tinospora cordifolia* (Amrutha balli) (Supplementary Figure 1). Prior to isolation, the selected fruits were first washed using 70% (v/v) ethanol, and sterile demineralized water for 2–3 min. About 1 g of the fruit pulp was suspended in 0.1 M phosphate-buffered saline (PBS) and homogenized to obtain a slurry. 1 mL of this slurry was enriched in 9 mL de Mann, Rogosa Sharpe (MRS) broth at 37 °C under anaerobic conditions for 48 h in multiple replicates. Following enrichment, 1 mL of this mixture was serially diluted (up to 10^–10^) in 0.1 M PBS and plated onto MRS agar plates. After incubation at 37 °C under anaerobic conditions for 48 h, morphologically distinct and pinpoint colonies were randomly selected and were repeatedly streaked onto MRS agar plates until pure colonies were obtained.

#### 2.1.2 Preliminary screening of LAB for antifungal activity

**FIGURE 1:**
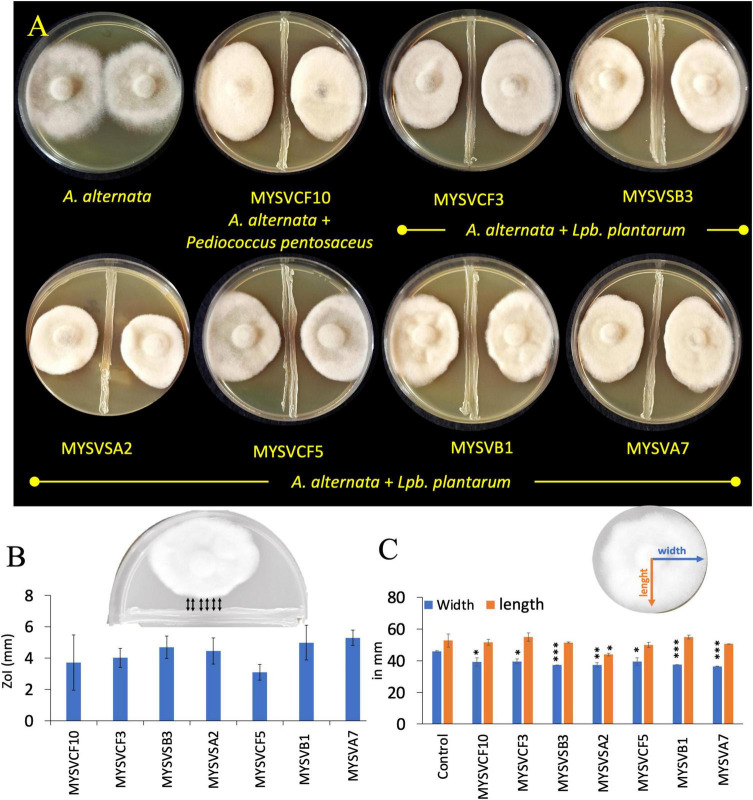
*In vitro*, dual confrontation assays showing the inhibition of the growth of *Alternaria alternata* by isolated LAB strains from various fruits **(A)**. The distinct area devoid of fungal growth between the LAB and *A. alternata*, were identified as the zone of inhibition (ZoI) **(B)**. Inset **(B)** provides a depiction of ZoI measurement highlighted in black arrows. Changes in the morphology of *A. alternata* were assessed by quantifying alterations in colony length and width in the presence of LAB **(C)**. Inset **(C)** features blue and orange lines representing width and length measurements. The x-axis in panels **(B,C)** represents the strain identity. Statistical significance at *p ≤ 0.05, ^**^p ≤ 0.01, ^***^p ≤ 0.001.

To evaluate the inhibitory activity of the isolated LAB against *A. alternata*, a dual confrontation assay was conducted with slight modifications (Gkarmiri et al., 2015). For this, overnight-grown isolates were first streak-plated in the center of the agar plate forming an 8.5 cm line. Subsequently, a 5 mm diameter fungal plug from the edge of an actively growing colony of *A. alternata* was placed on either side of the bacterial streak line. Control plates containing only *A. alternata* were also prepared. After 5 days of incubation at room temperature, the plates were examined for the growth of *A. alternata* and zone of inhibition (ZoI) around the isolates. The ZoI was defined by the distance, in millimeters, between the LAB and *A. alternata* (Figure 1B inset). The changes in the morphology of *A. alternata* in the presence of LAB were quantified by increase/decrease in the length and width of the colony with reference to control (Figure 1C inset).

### 2.2 Molecular identification of selected LABs having antifungal activity

Genomic DNA from overnight-grown LAB strains was extracted using GeNei™ kit following the manufacturer’s instructions. The 16S region of rDNA was amplified using universal primer sets: 8F-5′AGAGTTTGATCCTGGCTCAG 3′ and 1391 R-5′ GACGGGCGGTGWGTRCA 3′ (Somashekaraiah et al., 2021). The obtained amplicon was sequenced at Eurofins PVT. Ltd. Following identification, the sequences were submitted to the GenBank database to obtain accession IDs. A phylogenetic tree was constructed to further understand the evolutionary relationship between the identified LAB strains and the related species from the NCBI database. The tree was constructed by neighbor-joining method with a bootstrap value of 1,000 repetitions using MEGA 11 software.

### 2.3 Bacterial growth parameters

The LAB isolates were grown in MRS broth for 24–48 h at 37°C. After incubation, the cells were serially diluted and plated on MRS agar plates. The bacterial viability was calculated using the plate count method and expressed as logCFU.mL^–1^ (Reuben et al., 2019). Alternatively, the optical density at 600 nm at every hour was also measured to evaluate the growth of LAB.

Average division rate (R), the number of generations related to the growth time of the population was determined by *R* = (1/log2)x(logN_*t*_-logN_0_/t-t_0_). To determine the generation time (τ) or doubling time, the data points corresponding to the exponential phase of the growth curves were utilized in the following formula: *N = [log(N_*t*_)-log(N_0_)/log2].* Where log(*N*_0_) and log(*N*_*t*_) represent the logarithm of the number of cells at early and later time points during the exponential phase, respectively. The variable *N* signifies the number of generations within the specified time frame. Subsequently, the generation time (τ) was calculated using the formula: τ = *T/N* where, *T* denotes the time interval between *N*_0_ and *N*_*t*_, while *N* stands for the number of generations determined by the previous formula. The specific growth rate (μ), growth rate per unit of CFU was calculated using the formula: μ = 2.3[logN_*t*_-logN_0_/t-t_*o*_] (Kushkevych et al., 2019). The acidifying capacity was calculated as a function of the decrease in pH values during the growth (Rodríguez et al., 2021). The pH was determined at intervals of 1 h. Maximum acidification was determined as the lowest pH observed during the bacterial growth period. while acidification rate is characterized as the rate of change in pH within a specified time interval. For instance: ΔpH/ Δt = (pH_*t*_-pH_0_/t_1_-t_0_) (Rodríguez et al., 2021).

### 2.4 Probiotic attributes of selected LAB

The LAB isolates were initially characterized for phenotypic and biochemical traits, including Gram staining, catalase activity, bile salt hydrolase activity, osmotic tolerance (ranging from 3 to 7% NaCl), and carbohydrate fermentation (glucose, lactose, sucrose, xylose, maltose, D-arabinose, sorbitol, and D-raffinose) according to Cappuccino and Sherman (1999), Hedberg et al. (2008), and Shehata et al. (2016). The viability of the LAB strains was determined by calculating the logarithm of colony-forming units (CFU) per milliliter (mL) on MRS agar.

The LAB strains were grown in MRS broth under the anaerobic condition at 37°C for 16–20 h (∼7 log CFU/mL). The cell suspension was centrifuged at 8,000 rpm for 10 min, washed, and resuspended in 0.1M PBS, pH 7.4. The cell-pellet or resuspended pellet in PBS was used in the following assays unless specified otherwise.

#### 2.4.1 Cell surface hydrophobicity and auto-aggregation

To a 5 mL bacterial suspension in PBS, an equal volume of xylene was added, and mixed thoroughly. The mixture was allowed to stand undisturbed for 30 min, after which the absorbance of the aqueous phase measured at 600 nm. Percent hydrophobicity was calculated as [(A_0_-A_*t*_)/A_0_]x100, where A_*t*_ and A_0_ represent absorbance at 600 nm at a given time interval and at time 0, respectively.

To determine auto-aggregation, the cellular suspension in 0.1 M PBS was transferred to a glass cuvette. The change in optical density at 600 nm was measured every 1h intervals in a UV-Vis spectrophotometer. Percent aggregation at each time was calculated as [1-(A_*t*_/A_0_) x 100].

#### 2.4.2 Tolerance to temperatures and osmotic stress

About 50 μL of overnight grown active cell culture (∼7 log CFU/mL) was inoculated to a 5 mL previously salt-amended sterile MRS [3–7% (w/v)] and incubated at 37°C for 24 h. For assessing the growth at different temperatures, 50 μL of active cell culture was used to inoculate sterile MRS broth. The cultures were subsequently incubated for 24 h at 4, 10, 30, 37, and 60°C (Reuben et al., 2019). After incubation, 0.1 mL of the culture suspension was serially diluted and plated on MRS agar plates. The plates were incubated at 37°C for 24 to 48 h. The growth or viability was calculated as colony-forming units: (log.CFU.T_*t*_/ log.CFU.T_0_) x100. Where T_*t*_ is the viable count after incubation and T_0_ is the initial viable count.

#### 2.4.3 Tolerance to low pH, bile salt, and phenol

To assess growth and tolerance at varying low pH levels, the washed cell pellet was resuspended in 5 mL of previously pH-adjusted sterile MRS broth. pH values of 2, 3, and 4 were achieved by adding 1 N HCl to the initial pH 6. For bile salt tolerance, the cell pellet was resuspended in sterile MRS broth (Hi media, India) supplemented with 0.3% (w/v) bile salt (ox gall) (Loba Chemie, India). Alternatively, for the phenol tolerance test, the isolates were grown in MRS broth supplemented with 0.3–0.6% phenol (Adithi et al., 2022).

The LAB cultures under various experimental conditions were incubated for 1–4 or 24 h under anaerobic conditions at 37°C. At each 1 h time interval, the cultures were serially diluted and plated on MRS agar plates to determine the bacterial viability as described above.

### 2.5 Safety evaluation of the selected LAB

#### 2.5.1 Hemolytic activity

Blood agar plates were prepared using nutrient agar (Hi Media, India) supplemented with 5% (v/v) blood. The active LAB isolates were streak-plated forming a 3 cm line at the center of the plate. After 48 h of incubation at 37°C, the plates were examined to detect the presence of a distinct hemolysis pattern around the LAB colonies. The plates were classified as clean (β-hemolysis), greenish (α-hemolysis), and no hemolysis (γ-hemolysis). *Staphylococcus aureus* (ATCC 6538) served as a reference strain for hemolysis (Vanitha et al., 2023).

#### 2.5.2 Antibiotic susceptibility

The susceptibility of LAB strains to antibiotics was determined following Charteris et al. (1998) with minor modifications. The selected LAB isolates (100 μL) were spread-plated on MRS agar plates and air-dried aseptically. Antibiotic discs containing 2 μg/ disc of clindamycin, 10 μg/disc each of ampicillin, gentamycin, streptomycin, 15 μg/disc erythromycin, and 30 μg/disc each of vancomycin, kanamycin, chloramphenicol, and tetracycline were placed on inoculated plates. After 48 h of incubation at 37°C, the plates were examined for the zone of inhibition. The interpretive criteria for breakpoints were based on potential *Lactobacillus* sp. as outlined in Charteris et al. (1998) (Table 1).

**TABLE 1:** Antibiotic sensitivity of selected LAB isolates from tropical fruits.

Antibiotics (μg/disc)		Concentration (μg/disc)	*Lpb. plantarum*		Breakpoint criteria (mm)*		
			MYSVA7	MYSVB1	*R*	MS	*S*
Ampicillin	Amp	10	S	S	≤12	13–15	≥16
Clindamycin	Cli	2	S	S	≤8	9–11	≥12
Chloramphenicol	Chl	30	S	S	≤13	14–17	≥18
Erythromycin	Ery	15	S	S	≤13	14–17	≥18
Kanamycin	Kan	30	MS	MS	≤13	14–17	≥18
Streptomycin	Ste	10	MS	MS	≤11	12–14	≥15
Tetracycline	Tet	30	S	S	≤14	15–18	≥19
Vancomycin	Van	30	R	R	-	-	15

*Antibiotic susceptibly expressed as R (Resistant), MS (Moderately Susceptible) and S (Susceptible). The interpretive criteria for breakpoints of disc diffusion for potential *Lactobacillus* species as outlined in Charteris et al. (1998).

### 2.6 Antibacterial activity

The antibacterial properties of LAB isolates and their CFS against specific indicator bacteria: *Escherichia coli* (ATCC 25922), *Pseudomonas aeruginosa* (ATCC 15422), *Salmonella paratyphi* (ATCC 27820) and *Staphylococcus aureus* (ATCC 27870) was evaluated. For this, LAB cells and their CFS (both treated and untreated) were incubated with the indicator organisms. A part of the extracted CFS was neutralized to pH 7 (n-CFS) or proteinase K (1 mg.mL^–1^) treated (pCFS), or heat treated (100°C, 15 min) (hCFS) before use in the experiments (Rao et al., 2019).

### 2.7 Antifungal activity of LAB cells by co-inoculation assay

Experiments were conducted in a 100 mL Erlenmeyer flask containing 50 mL of modified MRS broth. Modified MRS broth was prepared by dissolving the following in demineralized water: 5 g/L Bacteriological peptone, 5 g/L mycological peptone, 5 g/L beef extract, 10 g/L yeast extract, 5 g/L dextrose, 0.1 g/L MgSO_4_, 0.05 g/L MnSO4, 2 g/L K_2_HPO_4._ The modified MRS was devoid of antifungal substances such as polysorbate 80, ammonium citrate and sodium acetate (Deepthi et al., 2016). Each flask was inoculated with 100 μL of LAB isolates and 100 μL of *A. alternata* (10^6^ spores/mL) and incubated at 37°C for 3, 7, 10, and 14 days. At the end of each pre-determined time interval, the mycelial biomass was harvested by filtration using Whatman no. 1 filter paper and dried at 60°C for 24 h. Simultaneously, the bacterial population in terms of log CFU/mL was determined by plate count technique. The flasks with bacterial cells and the spores of *A. alternata* alone served as control.

### 2.8 Antifungal activity of CFS of LAB isolates

#### 2.8.1 Biomass inhibition

Experiments were conducted in a 100 mL Erlenmeyer flask, each containing 50 mL of different ratios of CFS (5 to 20%, v/v). The desired CFS ratio was achieved by directly mixing CFS and sterile potato dextrose broth. Subsequently, *A. alternata* fungal discs with a diameter of approximately 7.5 mm were introduced into these flasks, followed by incubation at room temperature. Flasks without CFS served as control. After 10 days, the fungal mat was harvested as described above by filtration. The dry weight of each treatment was then compared to that of the control.

#### 2.8.2 Conidial germination inhibition

The experiments were conducted in a 24-well microtiter plate, using either LAB isolates or their CFS. In each well, 100 μL of LAB culture (∼10^6^ CFU/mL) and 100 μL of *A. alternata* spores (10^6^ spores/mL) in a 1:1 ratio were combined to reach a final volume of 1 mL, using 0.1 M PBS at pH 7.4. For the CFS, a mixture of 200 μL of CFS and *A. alternata* spores (10^6^ spores/mL) was prepared. The microtiter plate was then placed in an incubator at 30°C for 24 to 48 h. At each 2 h time interval, the germination of conidia was observed using a phase contrast microscope (Carl Zeiss AXIO, Germany). Briefly, 10 μL of conidial suspension (in triplicates) was transferred to a hemocytometer and directly observed under the microscope without any staining with brightfield illumination. The percentage of conidia germinated was determined by counting in a hemocytometer and calculated using the formula: Percentage = (Number of germinated conidia / Total conidia counted) x 100.

### 2.9 Stability and activity of CFS of LAB isolates

The stability of the CFS to inhibit *A. alternata* following proteinase K, pH neutralization, and high-temperature treatments was evaluated. For proteinase K, the CFS was treated with proteinase K (1 mg mL^–1^) at 37°C for 2 h, followed by enzyme inactivation at 80°C for 10 min. As for evaluating thermal stability, the CFS was heated to 100°C for 15 min and then cooled to room temperature. For the effect of pH, the CFS was neutralized to pH of 7 with 1 M NaOH. The pre-treated CFS was relabeled as heat-deactivated CFS (hCFS), proteinase K treatment (pCFS), and pH-neutralization (nCFS). The residual antifungal activity was measured in triplicate after each treatment in a 24-well microtiter plate, as detailed below. CFS shelf-life test was also conducted to evaluate the stability and antifungal activity after long storage and freeze-thaw conditions. The extracted crude CFS was stored at −20 or 4°C for >8 months. After which, conidial germination assay was conducted using the stored CFS as detailed above.

### 2.10 Identification of antifungal compounds

The CFS was recovered from LAB isolates after 72 h in modified MRS broth by centrifugation at 8,000 rpm for 15 min. The obtained supernatant was filter sterilized using a 0.22 μm syringe filter. Following this, the CFS was extracted using methanol and resuspended in the mobile phase before injection to Liquid chromatography with tandem mass spectrometry (LC-MS). For LC-MS analysis, the UPLC BEH-amide column was used at 25°C using organic and aqueous phases as mobile phases at 0.1 mL/min flow rate. The mobile phase consisted of 10 mM ammonium citrate and acetonitrile (1:1), pH 8.5. The initial flow conditions included 100% aqueous phase for 30 s, after which the gradient was changed to the ratio of 95:5 aqueous and organic phases for 6 min. After separation, the eluant from the column was directly pumped to a tandem quadrupole mass detector (TQD-MS/MS) without split for quantification of organic acids (Oliveira et al., 2008).

### 2.11 Microdilution method to evaluate the antifungal activity of succinic and lactic acids

The experiments were conducted in a 24-well microtiter plate, using different concentrations of organic acids in PDA broth. To each well, succinic acid (0–3 mg/mL), lactic acid (0–9 mg/mL), or a combination of succinic (0–1 mg/mL) and lactic acid (0–5 mg/mL) were added to the final volume of 2 mL. *A. alternata* plugs, measuring 3 mm in diameter, were introduced into each well. Wells with PDA alone, CFS, *A. alternata* plugs were considered as control. The plate was incubated at 30°C for 5 days. After incubation % inhibition was calculated as (biomass in control-biomass in treated/ biomass in control × 100). The Inhibitory Concentration (IC50) is determined as the concentration at which fungal growth is reduced by 50% compared to the control in the absence of an antifungal agent, in this case, organic acids or CFS (Pierce et al., 2008).

### 2.12 Statistical analysis

Experiments were conducted in a minimum of three replicates. The average and standard deviation from the replicates were used to plot the graphs in origin. The effect of LAB or its CFS extracts on *A. alternata* was determined using a student’s *t*-test. ANOVA was used to determine the variations in growth at different low pH and in antibacterial assays. Statistical analysis was performed using Microsoft Excel 2019. Statistical significance was determined at *p ≤ 0.05*.

## 3 Results

### 3.1 Isolation and screening of LAB from tropical fruits for antifungal activity

Lactic acid bacteria was successfully isolated from seven types of fruits. An average of 10^5^ CFU per g of fruit was obtained on MRS agar plates. Colonies that were pinpoint/small, circular, opaque, and displayed either white or colored morphological characteristics were selected for further screening and identification. After preliminary screening for negative catalase and positive gram tests, a total of fifty-five isolates were obtained with the highest counts (14) for *Solanum nigrum* and the lowest counts (4 each) for *Musa* and *Couroupita guianensis*.

Of the fifty-five LAB isolates obtained, only seven isolates exhibited inhibition against *A. alternata* (Figure 1). The inhibition was evident as irregular but compact growth near the area of LAB confrontation. The inhibitory zones produced by the LAB strains varied between 2- and 6-mm. Strains MYSVSB3 and MYSVA7 exhibited the highest inhibitory zones at >5.5 mm, while the strain MYSVCF5 exhibited the lowest at 2.3 mm among the seven isolates (Figures 1A, B). To ascertain the influence of LAB on the changes in the growth of *A. alternata*, the length, and width of the fungal colony were also measured (Figure 1C). All seven strains showed a significant reduction in the width of the fungal colony (*p* < 0.01), with the highest reduction with strains MYSVSB3, MYSVB1, and MYSVA7. Although the height or the length differed it was not statistically significant except for strain MYSVSA2 (*p* < 0.05). Strain MYSVSB3 did not show consistent results. Hence, it was screened out from further studies. Strains MYSVB1 and MYSVA7 showed promising and consistent inhibition against *A. alternata*. Moreover, a constriction in the morphology of the fungal colony was observed with these two strains (Figure 1A). Therefore, further studies were conducted with strains MYSVB1 and MYSVA7.

### 3.2 Molecular identification and growth kinetics of LAB from fruits

**FIGURE 2:**
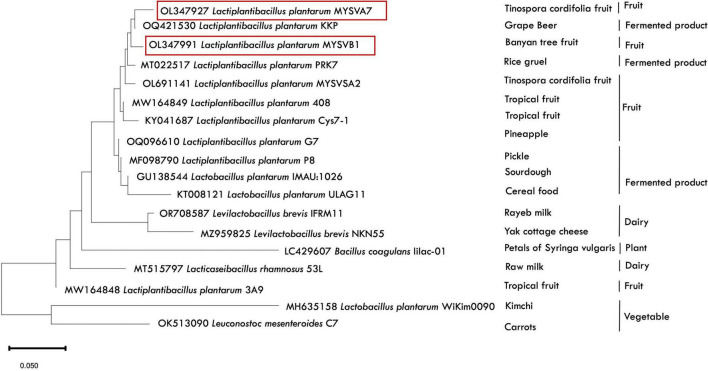
Phylogenetic analysis depicting the relative position of LAB isolates (MYSVA7 and MYSVB1) in comparison to other previously reported strains. The tree was built using 16S rDNA sequences (available in NCBI database) in Mega 11 software using maximum parsimony analysis. Strains isolated in this study are highlighted in red, with the source of isolation specified next to the strain identity. The alphanumerical (OL347927) represent the NCBI accession IDs for reference.

**FIGURE 3:**
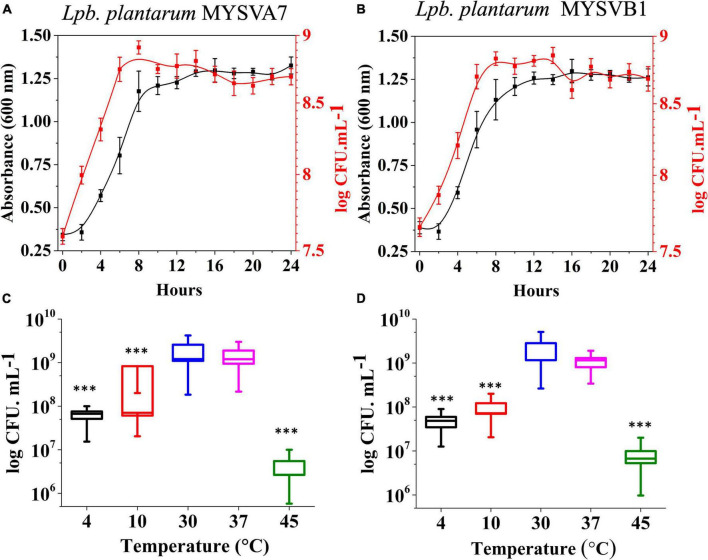
Growth kinetics **(A,B)** and growth at different temperatures **(C,D)** of strains *Lactiplantibacillus plantarum* MYSVA7 **(A,C)** and MYSVB1 **(B,D)**. The growth kinetics was studied at 37°C **(A,B)**. Statistical significance at ^***^p ≤ 0.001.

The 16S rRNA sequence of seven LAB isolates positive for antifungal activity was successfully obtained and analyzed. The genomic sequences confirmed the identity of six strains belonging to *Lactiplantibacillus* and one strain belonging to the *Pediococcus* genus. Each of the 16S rDNA sequences from these strains was assigned accession IDs. The identities, encompassing genus species and strain are *Pediococcus pentosaceus* MYSVCF10 (GenBank ID: OP563857), *Lpb. plantarum* MYSVCF3 (GenBank ID: OL347999), *Lpb. plantarum* MYSVSB3 (GenBank ID: OL691142), *Lpb. plantarum* MYSVSA2 (GenBank ID: OL691141), *Lpb. plantarum* MYSVCF5 (GenBank ID: OP563855), *Lpb. plantarum* MYSVB1 (GenBank ID: OL347991), and *Lpb. plantarum* MYSVA7 (GenBank ID: OL347927). With the selected *Lpb. plantarum* strains MYSVA7 and MYSVB1, a phylogenetic tree was constructed to visualize the relationship within the *Lactiplantibacillus* genus and other LABs reported in the literature (Figure 2). Based on sequence similarity and phylogenetic analysis, the next closest match was found to be *L. brevis* for isolates MYSVB1 and MYSVA7 (Figure 2). The strains MYSVA7 and MYSVB1 could be morphologically described as rods, non-spore-forming bacteria. Both the strains were able to ferment all the sugars without the formation of gas (Table 2). The growth kinetics of LAB strains MYSVA7 and MYSVB1 are shown in Figure 3. With the lag time of 2 h, the average division rate was observed to be 0.83 and 1 generation per h, respectively. Further, the LAB isolates’ acidification coincided with the growth due to the ability to ferment sugars and production of organic acids (data not shown). A strong association was observed between the growth rate and acidifying capacity. As the growth rate increased, the pH level of the medium decreased, ultimately reaching pH < 4 (Supplementary Table 1). Previous studies have established time required for fermentation of fruits and vegetables by autochthonous bacteria between 8 and 24 h (Di Cagno et al., 2013; Rodríguez et al., 2021). The maximum acidification in this study was obtained at time 10 h, and the maximum specific growth rate of 0.57 and 0.73 for strains MYSVB1 and MYSVA7 respectively (Supplementary Table 1). LAB strains were also evaluated for growth and cell viability at different temperatures. Variability of the growth kinetics was evident among the LAB strains. Both the strains exhibited the highest growth at >30°C. Although slight differences in the growth between 30 and 37 °C were observed, it was statistically insignificant (*p > 0.05*) for both temperatures. Therefore, the optimal temperature for LAB was >30°C. Growth at suboptimal temperatures at 10 and 4°C displayed a reduced growth (*p < 0.001*). An average reduction of >1 log CFU/ mL was observed for both temperatures (Figures 3A, B). Similar results were observed at high-temperature tolerance at 45 °C. In this case, a reduction by > 2 log CFU/mL (*p < 0.001*) was observed (Figures 3C, D).

**TABLE 2:** Carbohydrate fermentation ability of selected LAB isolates from fruits.

Carbohydrate fermentation	LAB isolates	
	MYSVA7	MYSVB1
Glucose	+	+
Lactose	+	+
Sucrose	+	+
Xylose	+	+
Maltose	+	+
D-Arabinose	+	+
Sorbitol	+	+
D-Raffinose	+	+

### 3.3 Probiotic attributes of selected LABs

#### 3.3.1 Cell surface properties

To evaluate the adhesive property, the cell surface hydrophobicity was measured. Notably, the hydrophobicity of the isolates exhibited consistency across the strains, at 25% (±3) for MYSVA7 and 28% (±2) for MYSVB1. Moreover, there was a discernible increase in percent aggregation over time. (Supplementary Figure 2). Particularly, both strains exhibited 25% aggregation after 5 h. Following a 24 h incubation, a further increase in aggregation index was observed, with percentages of 53 and 68%, respectively.

#### 3.3.2 Survival ability to osmotic stress and simulated gastrointestinal conditions

**FIGURE 4:**
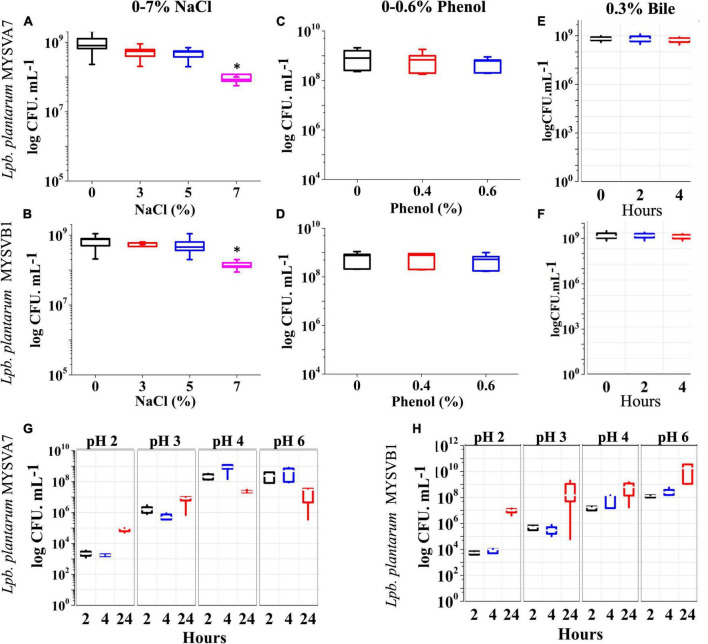
Probiotic properties of *Lactiplantibacillus plantarum* strains isolated from tropical fruits. The growth of LAB was assessed under various conditions, including different salinity levels (0–7% NaCl) **(A,B)**, phenol concentrations (0–0.6%) **(C,D)**, bile salt concentrations (0–0.3%) **(E,F)**, and varying pH conditions (pH 2–6) **(G,H)**. The properties for strains MYSVA7 are presented in panels **(A,C,E,G)**, while those for MYSVB1 are shown in panels **(B,D,F,H)**. Statistical significance at *p ≤ 0.05.

Figures 4A, B shows the tolerance of LAB isolates to different concentrations of salt. Both the isolates were tolerant to salt concentrations up to 7%. The cell viability was not affected by up to 5% salt concentration. An increase in salt to 7%, decreased the viability by 1 log CFU/mL (*p* < 0.05). On the other hand, with up to 0.6% phenol (Figures 4C, D) or 0.3% bile (Figures 4E, F), the viability of both strains was not compromised. The cellular viability of both strains was maintained above 9 log CFU/mL demonstrating >95% viability. Further, the LAB strains under simulated gastrointestinal conditions exhibited high tolerance, with minimal loss in viability (Figures 4G, H). Both strains MYSVA7 and MYSVB1 showed tolerance to pH 2. The cell counts of strain MYSVA7 at pH 3 and pH 2 after 4 h showed only 4 to 6 log CFU/mL as compared to pH 4 and above at 8 log CFU/mL. However, after 24 h, the cell counts reached 5 to 6 log CFU/mL at pH < 3 (Figure 4G). This was attributed to the combination of acidic pH and extended lag phase. No significant reduction (*p* > 0.05) was observed for strain MYSVA7 at pH < 3. Similar cell counts were observed for strain MYSVB1. At pH < 3, after 4 h, the viability decreased to 4 log CFU/mL from 7 log CFU/mL. However, after 24 h, the cell count increased to 7 log CFU/mL suggesting high tolerance and growth even at pH as low as 2 (Figure 4H). Overall, the isolated LAB showed high tolerance to osmotic stress and simulated gastrointestinal conditions.

#### 3.3.3 Antibacterial activity

**FIGURE 5:**
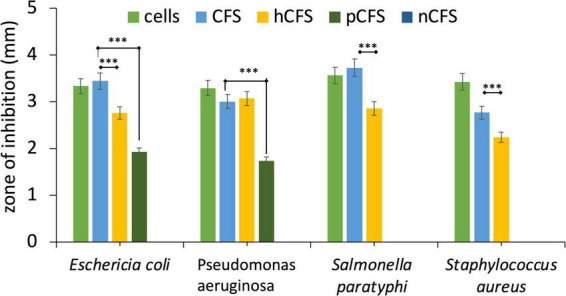
Antibacterial activity of *Lactiplantibacillus plantarum* MYSVB1. The target pathogen was inoculated with both viable cells of *Lpb. plantarum* MYSVB1 or its crude CFS. The CFS was pre-treated: heat-deactivated CFS (hCFS), proteinase K treated CFS (pCFS), and pH-neutralized CFS (nCFS) before use in the experiment. Statistical significance at ^***^p ≤ 0.001.

The antibacterial effectiveness of the strain MYSVB1 and its CFS was also evaluated against various pathogens including ESKAPE pathogens. The zone of inhibition for *E. coli*, *P. aeruginosa*, *S. paratyphi*, and *S. aureus* are shown in Figure 5. Notably, the cells of MYSVB1 and its crude CFS exhibited the highest inhibition against all the tested pathogens. The zone of inhibition ranged from 3 to 3.6 mm. However, antibacterial activity significantly declined after heat treatment (*p* < 0.001) (Figure 5). While treatment of proteinase K reduced the inhibition significantly (*p* < 0.05) for *E. coli*, *P. aeruginosa*, *S. paratyphi*, and *S. aureus* did not show any inhibition for growth (Figure 5). No inhibition was observed for CFS at pH 7 to any tested pathogens.

#### 3.3.4 The safety aspect of LABs

Both strains MYSVA7 and MYSVB1 showed no clear zone formation on blood agar. *Staphylococcus aureus* showed complete lysis of red blood cells evident by yellow coloration around its colony suggesting β-hemolysis (Supplementary Figure 3). Differences in susceptibility and resistance to common antibiotics were observed. Both strains showed similar sensitivity (Table 1). The strains were susceptible to ampicillin, clindamycin, chloramphenicol, erythromycin, and tetracycline. While kanamycin and streptomycin showed moderate sensitivity and complete resistance was observed with vancomycin. Nevertheless, the isolated strains were non-hemolytic and were susceptible to most of the common antibiotics tested.

### 3.4 Antifungal activity

**FIGURE 6:**
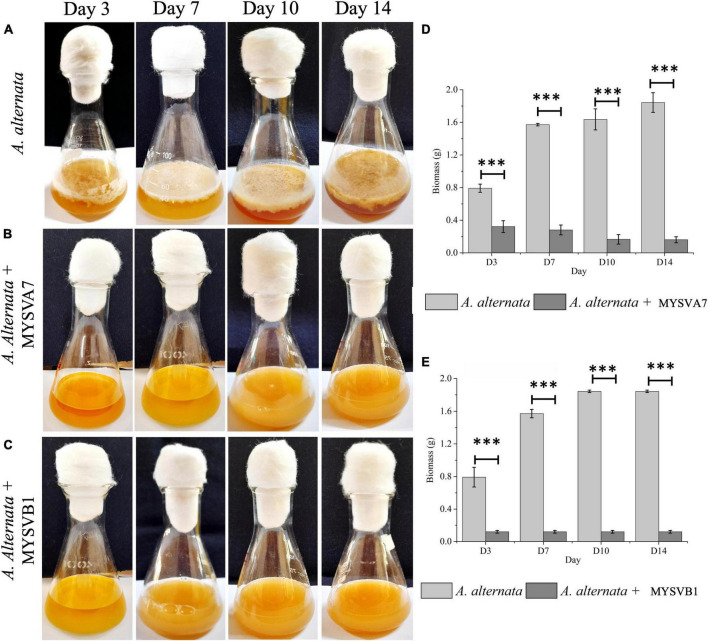
Co-inoculation assay demonstrating the inhibition of *A. alternata* by strains *Lactiplantibacillus plantarum* MYSVA7 and MYSVB1 on different days **(A–C)**. Time course the growth of mycelial mat in control **(A)** and in the presence of *Lpb. plantarum* MYSVA7 **(B)** and *Lpb. plantarum* MYSVB1 **(C)** are shown. The corresponding biomass yield harvested on different days are shown in panels **(D,E)**. Statistical significance at ***p ≤ 0.001.

Figure 6 illustrates the growth of *A. alternata* over a 14-day period. By day 3, visible thick mat growth was observed which continued to increase until day 14. By day 10 the fungal mat reached its maximum growth and the density of the mat remained unchanged thereafter. The weight of biomass exhibited progressive growth from day 1 until day 10, before reaching 1.8 g (dry weight). However, the introduction of viable cells of MYSVA7 (Figure 6B) and MYSVB1 (Figure 6C) significantly inhibited (*p* < 0.001) the growth of *A. alternata* by 87 and 75%, respectively, by day 3 (Figures 6D, E). As a result, no observable expansion or growth of fungal biomass was observed. Notably, the viability of the strain MYSVA7 and MYSVB1 during the 14-day period in the presence of *A. alternata* was not affected but displayed an exponential growth, lasting 3 days followed by a gradual decline over time (data not shown). These findings clearly demonstrate the effectiveness of strains in suppressing the growth of *A. alternata*, while maintaining its viability.

**FIGURE 7:**
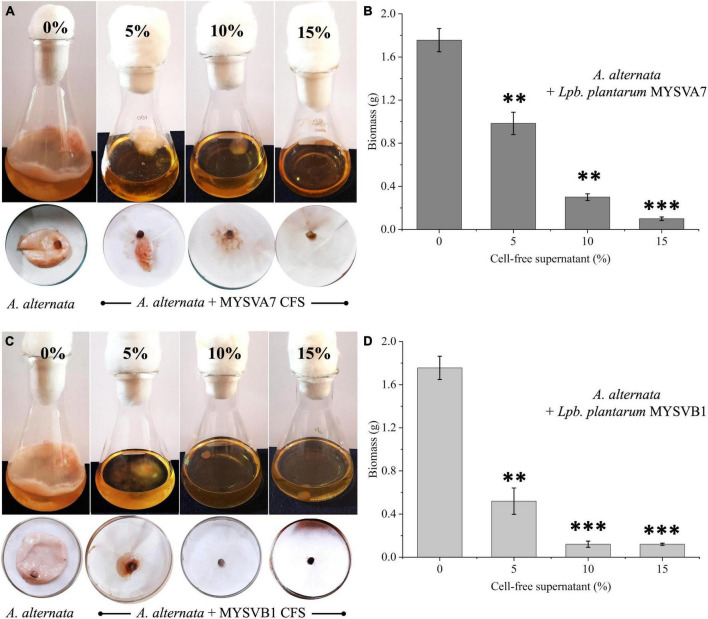
Antifungal activity of cell-free supernatant (CFS) extracted from *Lactiplantibacillus plantarum* MYSVA7 **(A,B)** and *Lpb. plantarum* MYSVB1 **(C,D)** against Alternaria alternata. Experiments were performed with different concentrations (%) of CFS. Flasks showing growth of *Alternaria alternata* with CFS of strain MYSVA7 **(A)** and MYSVB1 **(C)**. The corresponding fungal biomass yield harvested after 10 days of incubation at room temperature **(B,D)**. Statistical significance at **p ≤ 0.01, ***p ≤ 0.001.

Alternatively, when different ratios of crude CFS were used, differential inhibition was observed between the two strains (Figure 7). With just 5% of crude CFS, a significant inhibition (*p* < 0.01) in the growth of *A. alternata* was observed, with MYSVB1 displaying greater inhibition (*p* = 0.003). When the concentration of CFS from MYSVA7 was increased to 15%, the biomass yield of *A. alternata* decreased substantially by 94% (Figure 7B). Similarly, using only ≤ 10% CFS from strain MYSVB1 resulted in a comparable reduction in the yield. In fact, >70% decrease in biomass yield was recorded when only 5% of CFS of MYSVB1 was used (Figure 7D). Therefore, the CFS of strain MYSVB1 was considered the most potent inhibitor of *A. alternata*.

**FIGURE 8:**
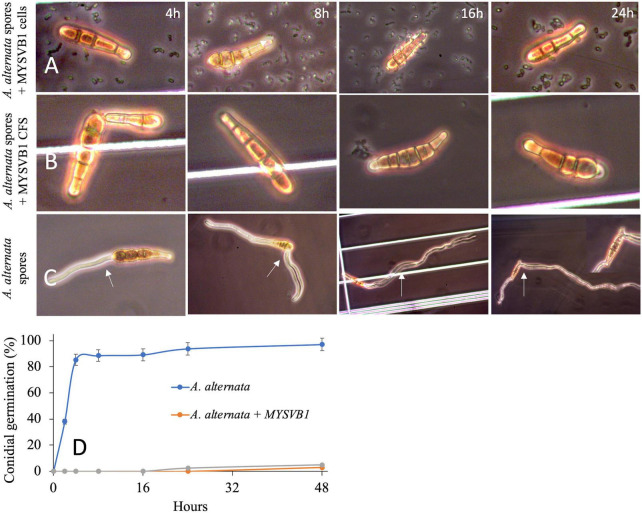
Germination of conidia of *Alternaria alternata* co-cultured with *Lactiplantibacillus plantarum* MYSVB1 **(A)** and its CFS **(B)** against control **(C)**. Panel **(D)** displays the corresponding time course of percentages of germinated conidia, measured at regular intervals. The white arrow shows the germination or formation of the germ tube.

The presence of viable cells of MYSVB1 had a pronounced inhibitory effect on both conidial germination and mycelial development. Microscopic examination revealed that even after 48 h, the germination of conidia impeded with viable cells or its CFS, with no observable germ tube formation (Figure 8). In striking contrast, the control groups exhibited rapid formation of germ tube within 4 h, followed by luxurious growth of mycelium (Figure 8A). By 16 h, 90% (of 10^6^ spores/mL) produced germ tubes, accompanied by the formation of mycelia (Figure 8B). It is worth noting that under nutrient-rich conditions, PDB medium, the germination of conidia and mycelial development occurred at an even faster rate (data not shown).

### 3.5 Tentative determination of antifungal substances

To identify the active components of CFS, following different treatments, antifungal assays were performed (Supplementary Figure 4). Heat treatment preserved the inhibitory properties, while pH neutralization and proteinase K treatment rendered them ineffective (Supplementary Figure 4). Both treatments showed no difference in inhibition compared to control without CFS, allowing fungal growth and sporulation. A pivotal role of the LAB’s acidic environment in its antifungal properties against *A. alternata*. Thus, the antifungal metabolites present in the culture filtrate (CFS) likely consist of acidic compounds, which may include organic acids and proteinaceous substances that are resistant to heat, possibly even a combination of components. With LC-MS, several organic acids were successfully identified in the CFS (Table 3). Specifically, lactic acid, pyruvic acid, succinic acid, malonic acid, fumaric acid, citric acid, hydroxy-citric acid, and shikimic acid were identified. Notably, Succinic acid was a predominant fraction, making up 63% (3.7 mg.mL^–1^) of the total organic acids identified, following lactic acid, and malic acid each at 9% (Table 3).

**TABLE 3:** Major organic acids composition of cell-free supernatant (CFS) of strain *Lpb. plantarum* MYSVB1.

Organic acids	Molecular weight (g.mol^−1^)	CFS (mg.mL^−1^)	% distribution
Succinic acid	118.09	3.7 ± 0.02	63.8
Lactic acid	90.08	0.5 ± 0.004	9.3
Malic acid	116.1	0.54 ± 0.02	9.2
Malonic acid	104.06	0.39 ± 0.02	6.6
Citric acid	192.12	0.38 ± 0.3	6.4
Pyruvic acid	88.06	0.12 ± 0.01	2.0

### 3.6 In vitro antifungal activity of organic acid

Succinic acid, identified as the predominant organic acid in CFS through LC-MS, was further investigated for its *in vitro* antifungal activity against *A. alternata*. As the concentration of succinic acid increased, a corresponding increase in percent inhibition was observed. At a concentration of 0.8 mg/mL succinic acid, mirroring the observed concentration in 20% CFS, the percent inhibition ranged from 38 to 40%. A further increase in concentration to > 2.5 mg/mL resulted in inhibition exceeding 90% (Supplementary Figure 4A). The IC_50_ for succinic acid was determined to be between 1.8 and 1.9 mg/mL, which was more than four times the concentration found in 20% CFS. Consequently, additional experiments were conducted to explore lactic acid, the second most prevalent organic acid in CFS.

With the addition of 0.1 mg/mL lactic acid, corresponding to the concentration in 20% CFS, no discernible differences were noted. Even with an increase in concentration to 0.6 mg/mL lactic acid in conjunction with 1 mg/mL succinic acid, no variations were observed (Supplementary Figure 4C). However, upon further increasing the concentration of lactic acid to 9 mg/mL, complete inhibition of *A. alternata* was achieved (Supplementary Figure 4B). The IC_50_ for lactic acid was determined to be >5 mg/mL. Consequently, the observed concentration of succinic acid in CFS alone could not replicate the inhibition observed in CFS, even when combined with lactic acid. Thus, succinic acid appears to contribute partially to the growth inhibition, possibly through a synergistic interaction with other organic acids.

## 4 Discussion

Enhancing crop yield through effective protection against plant pathogens has long been a pivotal objective in agriculture. *Alternaria* sp. is responsible for causing severe foliar disease, known as blight in several plants. Combatting the infection of *Alternaria* sp. is crucial for ensuring agricultural productivity (Stewart et al., 2013; Tanahashi et al., 2016). In this context, the role of beneficial microorganisms, such as LABs are important players. LAB, with its well-documented antimicrobial properties, can play a vital role in strengthening plant health and combating phytopathogens like *Alternaria*. Moreover, LABs have gained prominence as probiotics for fostering balance in the intestinal microbiota of humans and animals and for their ability to inhibit or fight against pathogenic or infectious diseases (Argyri et al., 2013). With growing interest in green alternatives for improved health, fruits and vegetables are an important source of probiotics (Di Cagno et al., 2010; Garcia et al., 2016; Zhang et al., 2022). As fruits are traditionally safe for consumption without requiring processing, LAB from fruits are natural choices for probiotics. In this study, a total of fifty-five isolates were obtained from seven different tropical and previously unexplored fruits. Of these LAB strains, seven strains showed antagonistic activity against phytopathogen *A. alternata*. Two strains, namely MYSVA7 and MYSVB1 showed sustained inhibition against *A. alternata*. The analysis revealed that these strains exhibited several characteristics morphological and biochemical traits such as the genus *Lactiplantibacillus* including Gram-positive, rod, catalase-negative, and non-spore-forming, and the tendency to aggregate and demonstrated facultative hetero-type fermentation. 16S rDNA sequencing confirmed this observation with *L. plantarum* having the highest (>99%) homology. To the best of our knowledge, this is the first report, to isolate and evaluate probiotic characteristics for tropical fruits having antifungal activity against a phytopathogen *A. alternata*.

The ability to maintain viability and adhere to is a determinant factor in many recommendations regarding probiotics (Zhang et al., 2022). The intestinal bile salts and the acidic environments of the stomach have been identified as the primary challenges to the survival of LAB within the GI tract (Hsu et al., 2018). The growth of MYSVA7 and MYSVB1 was not affected under conditions of salt up to 7% and temperature ranging from 4 to 45°C. Despite a minor growth delay at higher mesophilic temperatures (*T* = 45°C), the notable tolerance and adaptability exhibited by these two strains strongly support their capability for both high- and low-temperature fermentation. Moreover, under the condition of pH 2 to 6, and in the presence of bile (up to 0.6%) the growth was not affected. At pH 4–6, the pH of the fermentation broth decreased in pH value rapidly. It is widely documented that LAB can decrease the pH by the production of organic acids (Hsu et al., 2018). It is important to note that these strains displayed resilience within a broad pH range, from pH 2 to 6, and even in the presence of bile concentrations up to 0.6% bile. The differing levels of survivability in acidic conditions can likely be attributed to species- or strain-specific mechanisms of acid tolerance potentially involving specific bacterial proteins that confer resistance (Nami et al., 2019).

One of the hallmark characteristics of LABs is the ability for auto aggregation that allows the microorganisms of the same species to form cohesive groups, often binding to the intestinal mucosa (Lukic et al., 2014) or co-aggregation with other strains enabling interactions, potentially with pathogens serving as a host defense against infections (Goh and Klaenhammer, 2010). The strains in this study displayed varying degrees of auto-aggregation. However, with high tolerance to GI conditions and aggregation ability, the isolates could significantly contribute to intestinal health. Several studies have highlighted certain probiotic strains can potentially combat intestinal pathogens effectively (Kumar et al., 2010; Campana et al., 2017; Mirzaei et al., 2018). Considering growing concerns surrounding antibiotic resistance, the antimicrobial capabilities against pathogenic bacteria have gained significant attention (Lee et al., 2013). Within the isolates, the strains exhibited robust antibacterial activity. Notably, the CFS exerted the same efficacy of viable cells in inhibiting the pathogenic bacteria suggesting the production and release of antimicrobial substances. Moreover, the CFS could inhibit both Gram-positive and Gram-negative pathogens. LABs are known for their health-promoting attributes, beyond their antibacterial activity, which encompasses antiviral and potential anticancer properties (Drider et al., 2016; Kaur et al., 2017). While this study did not explore these specific aspects, it is an intriguing avenue for future research, where LAB’s broader therapeutic potential in the context of viral infections and cancer could be studied.

Despite the long history of safe usage of LAB as food-grade microorganisms, it remains essential to consider and assess the potential virulence-associated factors (Li et al., 2020). In this study, none of the isolated strains exhibited erythrocyte lysis confirming the safety aspect but does not confirm the absence of virulence factors. The strains were also susceptible to common antibiotics. Notably, both strains were resistant to vancomycin antibiotics. Vancomycin resistance within the genus *Lactiplantibacillus* is typically classified as intrinsic, primarily due to the peptidoglycan structure of the bacterial cell wall. Similar reports of probiotics from dietary supplements and dairy products also exhibited resistance to vancomycin, kanamycin, and gentamycin (Li et al., 2020). Overall, the isolates were deemed safe as potential probiotics. Following the antibacterial activity, the strains *Lpb. plantarum* MYSVA7 and MYSVB1 consistently demonstrated the most significant antifungal activity in both solid and liquid substrate-based models. It is worth noting that many species within the genus have been previously documented to exhibit antifungal properties in dairy products, silage fermentation, etc. (Li et al., 2020). Strains belonging to *L. plantarum* have shown inhibition against *Fusarium*, *Penicillium*, and *Aspergillus*, among others (Wang et al., 2012; Deepthi et al., 2016; Adithi et al., 2022). In this study, it’s worth noting that despite both strains belonging to *Lpb. plantarum* species, the antifungal activity displayed strain-specific variations. Specifically, MYSVB1 showed robust inhibition against *A. alternata*. This variance in antifungal efficacy observed in previous research was attributed to the strains’ potential to produce antifungal molecules, which might be limited by their ability to attain higher population levels (Leyva Salas et al., 2017). However, both strains displayed an optimal growing temperature between 30–37°C with comparable generation time (0.8–1 generation per h). These differences were consistent when crude CFS was used instead of viable cells. For instance, 5% CFS derived from strain MYSVB1 was sufficient to inhibit the fungal growth by 70% while it required ∼8–10% CFS from MYSVA7 to inhibit fungal growth by 70%. viz., strain MYSVB1 was more potent by at least a factor of two than MYSVA7 in inhibiting the growth of *A. alternata*. Further, the inhibitory effects on spore germination were also confirmed. Previous research has consistently shown that the antimicrobial compounds present in CFS produced by LAB tend to specifically target the cell membranes of pathogenic microorganisms. This interaction results in a range of effects, including changes in cellular morphology, and disruption of surface structures on conidia, ultimately damaging the cell membranes (Xu et al., 2019). The modulation of cell membrane permeability stands out as a fundamental antimicrobial mechanism employed by LAB. Zhao et al. (2022) suggested that intracellular substance leakage resulting from peroxidation damage to hyphae is a major mechanism of action. Although this is well-documented in different *Lactiplantibacillus* strains (He et al., 2020), the changes in the conidial morphology due to cells or its CFS were not observed in this study, but rather complete inhibition was observed.

The CFS of MYSVB1 demonstrated excellent thermostability, and stability in an acidic environment but poor enzymatic stability (Supplementary Figure 4). The antimicrobial properties exhibited by the CFS tend to remain across a wide range of temperatures. This is primarily attributed to the low molecular weight antimicrobial compounds (Girma and Aemiro, 2021). In addition to this, the loss of antifungal activity after protease treatment suggests the presence of proteinaceous substances in CFS. The present investigation confirmed succinic acid as the predominant antifungal molecule synthesized by MYSVB1. Notably, its accumulation was found to reach higher concentrations than MIC for bacteria at 320 μg/mL (Kumar et al., 2018) to inhibit the *A. alternata* spore growth effectively. Under anaerobic conditions, succinic acid and lactic acid serve as the primary fermentation products. Among the three possible routes for succinic acid production, the conversion of malic acid into succinic acid, facilitated by succinate dehydrogenase, is the most favorable reaction (Wang et al., 2021). Besides, lactic acid and malic acid are the second most abundant organic acids produced by the MYSVB1 strain. The generation of lactic acid may arise from the decarboxylation of malic acid through malolactic enzyme activity or the reduction of pyruvate by lactate dehydrogenase (Wang et al., 2021). Markedly, the absence of acetic acid and the presence of pyruvic acid suggest succinic acid production via the malic acid pathway. This insight adds depth to our understanding of the metabolic processes involved. However, further experiments are needed to confirm the actual pathway. When succinic acid was evaluated independently, the inhibitory concentration for succinic acid was determined to be approximately 1.9 mg/mL, which is twice the concentration identified in CFS. Nevertheless, when combined with a higher concentration of lactic acid, the inhibitory effect was observed to increase. Consequently, it can be concluded that succinic acid, at the observed concentration alone, only contributes partially to the antifungal activity. Succinic acid has already been described for its ability to cause loss of viability and cell destruction as a weak acid (Orij et al., 2012). The enhanced inhibition observed in combination with lactic acids suggests that a synergistic interaction among organic acid compounds providing a more comprehensive explanation for the observed activity. Moreover, the observed poor efficacy at both alkaline and neutral pH may be due to the dissociation of organic acids. Therefore, when contemplating the application in CFS, it is important to account for acid-base conditions of the food matrix. Nevertheless, CFS demonstrated robust stability and can be safely stored for extended durations, ranging up to 10 months, within a temperature range spanning from 4 to 35°C.

It is important to acknowledge that the observed antifungal activity is attributed to organic acids, specifically succinic acid. It is also suggested that the compound may also contain proteinaceous molecules. However, at this point, the proteinaceous content in the CFS could not be confirmed. For a comprehensive understanding of the applicability of *Lpb. plantarum* strain MYSVB1 or its CFS, it is essential to conduct further studies aimed at elucidating the antimicrobial components, factors influencing their stability, and *in vivo* assays in field conditions.

## 5 Practical implications

*Alternaria* disease has been a global problem since its first report of leaf blotch in apple trees in 1926. The disease is typically prevalent in regions with dry, warm weather conditions. But more recently it has emerged in temperate zones. *Alternaria* disease appears during warm weather and heat periods, with initial symptoms showing up in early summer. As the summer progresses and temperatures drop, the symptoms worsen, leading to severe defoliation and the appearance of fruit spots. This results in 85% defoliation and 80% infested fruits per orchard. Besides, *Alternaria* sp. can also cause other diseases like moldy core during postharvest storage. Therefore, the incidence of *Alternaria* disease can occur both during cultivation and post-harvest, leading to significant economic losses. Hence, effective strategies are necessary to manage *Alternaria*-related diseases for sustainable agriculture. *Alternaria* disease is traditionally managed by applying chemical fungicides through spray treatments. Few studies reported success in controlling the disease in various crops, including *Solanum tuberosum*, *Capsicum annuum*, sunflower, and Jerusalem artichoke (Viriyasuthee et al., 2019). However, due to evolving agricultural policies, aiming to reduce pesticide use by up to 50% by 2030 and the adverse effects of such fungicides, there is a need for innovative strategies. The use of resistant genotypes and biocontrol methods involving *Trichoderma* sp., however, have proven effective in peanuts, sunflower, chili, and aloe vera (Viriyasuthee et al., 2019) but not widely studied.

Using LABs as antifungal agents for crop protection is a promising prospect. LABs are naturally occurring microorganisms that have been found to offer a range of health benefits to humans, such as improving digestive health and boosting the immune system. As a probiotic, LABs can support the growth of beneficial bacteria in the gut, leading to improved overall health and wellbeing. By leveraging the natural properties of LABs, it is possible to protect the crops against harmful fungi while simultaneously promoting sustainable agricultural practices. The diverse population of LAB found in fruits can be a valuable resource for producing various probiotic products. The two novel LAB strains, isolated from *Tinospora cordifolia and Ficus benghalensis*: *Lpb. plantarum* MYSVA7 and *Lpb. plantarum* MYSVB1, respectively, offers an alternative solution against *A. alternata*. *Lpb. plantarum* MYSVB1 and its CFS showed imbibition of both mycelial growth and conidial germination. Moreover, it demonstrated in-vitro probiotic attributes, suggesting its potential as a probiotic strain. To ensure their suitability for industrial processing and storage, LAB strains, including the novel *Lpb. plantarum* MYSVB1 must undergo essential technological assessments. Further research should be focused on understanding their mechanisms of action and assessing their antifungal properties against other *Alternaria* sp. including the human pathogenic strains. In addition, it is important to study the *in vivo* efficacy of LAB as a probiotic and in-planta experiment of CFS as a biocontrol agent. This exploration has the potential to promote sustainable agricultural practices while capitalizing on the probiotic advantages offered by LABs.

## 6 Conclusion

A total of fifty-five LAB strains were isolated from seven different tropical fruits. Among these, seven isolates showed inhibition to *A. alternata*. Two strains, *Lactiplantibacillus plantarum* MYSVA7, and *Lpb. plantarum* MYSVB1, showed promise as probiotics isolated from *Tinospora cordifolia* and *Ficus benghalensis* fruits, respectively. These strains demonstrated robust probiotic characteristics, including resistance to acidic pH (pH 4–6), 0.3% bile salt, 0.6% phenol and osmolarity (3–7% salt), as well as antibacterial activities against selected pathogens. Notably, these strains exhibited antifungal activity against *Alternaria alternata*, with strain MYSVB1 showing stable inhibition. The crude cell-free supernatant (CFS) from MYSVB1 exhibited superior inhibition, achieving a 70% growth reduction of *A. alternata* at 5%, and > 95% at 10% CFS. Additionally, the CFS also suppressed the germination of conidia. The specificity of MYSVB1 in inhibiting *A. alternata* suggested the involvement of a combination of several organic acids and proteinaceous substances. Succinic acid was the predominant organic acid present in CFS at 63%. yet it partly contributed to the inhibition. Further studies are needed to identify the antifungal molecule in CFS and test its efficacy in-planta experiments. This study contributes to the knowledge of LAB diversity in tropical fruits and their potential applications in probiotics and biocontrol against fungal phytopathogens.

## Data availability statement

The data presented in the study are deposited in the National Center for Biotechnology Information (NCBI) repository and accession numbers can be found in the article.

## Author contributions

RV: Conceptualization, Data curation, Formal analysis, Methodology, Writing—original draft. MS: Data curation, Validation, Writing—review and editing. RS: Resources, Writing—review and editing. AG: Resources, Writing—review and editing. MYS: Supervision, Writing—review and editing.

## References

[B1] Adithi G. Somashekaraiah R. Divyashree S. Shruthi B. Sreenivasa M. Y. (2022). Assessment of probiotic and antifungal activity of *Lactiplantibacillus plantarum* MYSAGT3 isolated from locally available herbal juice against mycotoxigenic *Aspergillus* species. *Food Biosci.* 50:102118.

[B2] Argyri A. A. Zoumpopoulou G. Karatzas K. A. G. Tsakalidou E. Nychas G. J. E. Panagou E. Z. (2013). Selection of potential probiotic lactic acid bacteria from fermented olives by in vitro tests. *Food Microbiol.* 33 282–291. 23200662 10.1016/j.fm.2012.10.005

[B3] Armitage A. D. Cockerton H. M. Sreenivasaprasad S. Woodhall J. Lane C. R. Harrison R. J. (2020). Genomics evolutionary history and diagnostics of the *Alternaria alternata* species group including apple and Asian pear pathotypes. *Front. Microbiol.* 10:3124. 10.3389/fmicb.2019.03124 32038562 PMC6989435

[B4] Campana R. van Hemert S. Baffone W. (2017). Strain-specific probiotic properties of lactic acid bacteria and their interference with human intestinal pathogens invasion. *Gut Pathog.* 9:12. 10.1186/s13099-017-0162-4 28286570 PMC5338089

[B5] Cappuccino J. G. S. Sherman N. (1999). *Microbiology: a Laboratory Manual*, 10th Edn. : California, CA: Pearson Benjamin Cummings.

[B6] Charteris W. P. Kelly P. M. Morelli L. Collins J. K. (1998). Antibiotic susceptibility of potentially probiotic Lactobacillus species. *J. Food Prot.* 61 1636–1643. 9874341 10.4315/0362-028x-61.12.1636

[B7] Deepthi B. V. Rao P. Chennapa M. K. N. Chandrashekara K. T. Sreenivasa M. Y. (2016). Antifungal attributes of *Lactobacillus plantarum* MYS6 against Fumonisin producing *Fusarium proliferatum* associated with poultry feeds. *PLoS One* 11:e0155122. 10.1371/journal.pone.0155122 27285317 PMC4902316

[B8] Di Cagno R. Cardinali G. Minervini G. Antonielli L. Rizzello C. G. Ricciuti P. (2010). Taxonomic structure of the yeasts and lactic acid bacteria microbiota of pineapple (*Ananas comosus* L. Merr.) and use of autochthonous starters for minimally processing. *Food Microbiol.* 27 381–389. 10.1016/j.fm.2009.11.012 20227603

[B9] Di Cagno R. Coda R. De Angelis M. Gobbetti M. (2013). Exploitation of vegetables and fruits through lactic acid fermentation. *Food Microbiol.* 33 1–10. 23122495 10.1016/j.fm.2012.09.003

[B10] Drider D. Bendali F. Naghmouchi K. Chikindas M. L. (2016). Bacteriocins: not only antibacterial agents. *Probiotics Antimicrob.* 8 177–182. 10.1007/s12602-016-9223-0 27481236

[B11] Gabriel M. F. Uriel N. Teifoori F. Postigo I. Suñén E. Martínez J. (2017). The major *Alternaria alternata* allergen, Alt a 1: a reliable and specific marker of fungal contamination in citrus fruits. *Int. J. Food Microbiol.* 257 26–30. 10.1016/j.ijfoodmicro.2017.06.006 28633053

[B12] Gai Y. Niu Q. Kong J. Li L. Liang X. Cao Y. (2023). Genomic and transcriptomic characterization of *Alternaria alternata* during infection. *Agronomy* 13:809.

[B13] Garcia E. F. Luciano W. A. Xavier D. E. da Costa W. C. de Sousa Oliveira K. Franco O. L. (2016). Identification of lactic acid bacteria in fruit pulp processing byproducts and potential probiotic properties of selected *Lactobacillus* strains. *Front. Microbiol.* 7:1371. 10.3389/fmicb.2016.01371 27625647 PMC5003889

[B14] Girma A. Aemiro A. (2021). Antibacterial activity of lactic acid bacteria isolated from fermented Ethiopian traditional dairy products against food spoilage and pathogenic bacterial strains. *J. Food Qual.* 2021:9978561.

[B15] Gkarmiri K. Finlay R. D. Alström S. Thomas E. Cubeta M. A. Högberg N. (2015). Transcriptomic changes in the plant pathogenic fungus *Rhizoctonia solani* AG-3 in response to the antagonistic bacteria *Serratia proteamaculans* and *Serratia plymuthica*. *BMC Genom.* 16:630. 10.1186/s12864-015-1758-z 26296338 PMC4546130

[B16] Goh Y. J. Klaenhammer T. R. (2010). Functional roles of aggregation-promoting-like factor in stress tolerance and adherence of *Lactobacillus acidophilus* NCFM. *Appl. Environ. Microbiol.* 76 5005–5012. 10.1128/AEM.00030-10 20562289 PMC2916482

[B17] He J.-F. Jin D.-X. Luo X.-G. Zhang T.-C. (2020). LHH1, a novel antimicrobial peptide with anti-cancer cell activity identified from *Lactobacillus casei* HZ1. *AMB Express*, 10, 1–15. 10.1186/s13568-020-01139-8 33175275 PMC7658291

[B18] Hedberg M. Hasslöf P. Sjöström I. Twetman S. Stecksén-Blicks C. (2008). Sugar fermentation in probiotic bacteria–an in vitro study. *Oral Microbiol. Immunol.* 23 482–485. 10.1111/j.1399-302X.2008.00457.x 18954354

[B19] Hsu T. C. Yi P. J. Lee T. Y. Liu J. R. (2018). Probiotic characteristics and zearalenone-removal ability of a *Bacillus licheniformis* strain. *PLoS One* 13:e0194866. 10.1371/journal.pone.0194866 29641608 PMC5895015

[B20] Kaur M. Singh H. Jangra M. Kaur L. Jaswal P. Dureja C. (2017). Lactic acid bacteria isolated from yak milk show probiotic potential. *Appl. Microbiol. Biotechnol.* 101 7635–7652. 28879447 10.1007/s00253-017-8473-4

[B21] Kumar R. Chandar B. Parani M. (2018). Use of succinic & oxalic acid in reducing the dosage of colistin against New Delhi metallo-β-lactamase-1 bacteria. *Indian J. Med. Res.* 147 97–101. 29749367 10.4103/ijmr.IJMR_1407_16 PMC5967224

[B22] Kumar V. J. R. Seo B. J. Mun M. R. Kim C. J. Lee I. Kim H. (2010). Putative probiotic *Lactobacillus* spp. from porcine gastrointestinal tract inhibit transmissible gastroenteritis coronavirus and enteric bacterial pathogens. *Trop. Anim. Health Prod.* 42 1855–1860. 10.1007/s11250-010-9648-5 20623187 PMC7089342

[B23] Kumari V. B. Huligere S. S. Ramu R. Naik Bajpe S. Sreenivasa M. Y. Silina E. (2022). Evaluation of probiotic and antidiabetic attributes of *Lactobacillus* strains isolated from fermented beetroot. *Front. Microbiol.* 13:911243. 10.3389/fmicb.2022.911243 35774469 PMC9237538

[B24] Kushkevych I. Kotrsová V. Dordević D. Buňková L. Vítězová M. Amedei A. (2019). Hydrogen sulfide effects on the survival of lactobacilli with emphasis on the development of inflammatory bowel diseases. *Biomolecules* 9:752. 10.3390/biom9120752 31756903 PMC6995546

[B25] Lee J. S. Chung M. J. Seo J. G. (2013). In vitro evaluation of antimicrobial activity of lactic acid bacteria against *Clostridium difficile*. *Toxicol. Res.* 29 99–106. 24278635 10.5487/TR.2013.29.2.099 PMC3834449

[B26] Leyva Salas M. Mounier J. Valence F. Coton M. Thierry A. Coton E. (2017). Antifungal microbial agents for food biopreservation—a review. *Microorganisms* 5:37. 10.3390/microorganisms5030037 28698479 PMC5620628

[B27] Li M. Wang Y. Cui H. Li Y. Sun Y. Qiu H. J. (2020). Characterization of lactic acid bacteria isolated from the gastrointestinal tract of a wild boar as potential probiotics. *Front. Vet. Sci.* 7:49. 10.3389/fvets.2020.00049 32118070 PMC7026679

[B28] Lukic J. Strahinic I. Milenkovic M. Nikolic M. Tolinacki M. Kojic M. (2014). Aggregation factor as an inhibitor of bacterial binding to gut mucosa. *Microb. Ecol.* 68 633–644. 10.1007/s00248-014-0426-1 24823989

[B29] Maria, do Socorro M. R. Alves R. E. de Brito E. S. Pérez-Jiménez J. Saura-Calixto F. (2010). Bioactive compounds and antioxidant capacities of 18 non-traditional tropical fruits from Brazil. *Food Chem.* 121 996–1002.

[B30] Masunaka A. Ohtani K. Peever T. L. Timmer L. W. Tsuge T. Yamamoto M. (2005). An isolate of *Alternaria alternata* that is pathogenic to both tangerines and rough lemon and produces two host-selective toxins, ACT- and ACR-toxins. *Phytopathology* 95 241–247. 18943116 10.1094/PHYTO-95-0241

[B31] Mirzaei E. Z. Lashani E. Davoodabadi A. (2018). Antimicrobial properties of lactic acid bacteria isolated from traditional yogurt and milk against *Shigella* strains. *GMS Hyg. Infect. Control* 13:Doc01. 10.3205/dgkh000307 29416958 PMC5784316

[B32] Nami Y. Vaseghi Bakhshayesh R. Mohammadzadeh Jalaly H. Lotfi H. Eslami S. Hejazi M. A. (2019). Probiotic properties of *Enterococcus* isolated from artisanal dairy products. *Front. Microbiol.* 10:300. 10.3389/fmicb.2019.00300 30863379 PMC6400110

[B33] Oliveira A. P. Pereira J. A. Andrade P. B. Valentão P. Seabra R. M. Silva B. M. (2008). Organic acids composition of *Cydonia oblonga* Miller leaf. *Food Chem.* 111 393–399. 10.1016/j.foodchem.2008.04.004 26047441

[B34] Orij R. Urbanus M. L. Vizeacoumar F. J. Giaever G. Boone C. Nislow C. C. (2012). Genome-wide analysis of intracellular pH reveals quantitative control of cell division rate by pH in *Saccharomyces cerevisiae*. *Genome Biol.* 13:R80. 10.1186/gb-2012-13-9-r80 23021432 PMC3506951

[B35] Pierce C. G. Uppuluri P. Tristan A. R. Wormley F. L. Mowat E. Ramage G. (2008). A simple and reproducible 96-well plate-based method for the formation of fungal biofilms and its application to antifungal susceptibility testing. *Nat. Protoc.* 3 1494–1500. 10.1038/nport.2008.141 18772877 PMC2741160

[B36] Rao P. K. Deepthi B. V. Rakesh S. Ganesh T. Achar P. Sreenivasa M. Y. (2019). Antiaflatoxigenic potential of cell-free supernatant from *Lactobacillus plantarum* MYS44 against *Aspergillus parasiticus*. *Probiotics Antimicrob.* 11 55–64. 10.1007/s12602-017-9338-y 29064057

[B37] Reuben R. C. Roy P. C. Sarkar S. L. Alam R. U. Jahid I. K. (2019). Isolation, characterization, and assessment of lactic acid bacteria toward their selection as poultry probiotics. *BMC Microbiol.* 19:253. 10.1186/s12866-019-1626-0 31718570 PMC6852909

[B38] Rodríguez L. G. R. Gasga V. M. Z. Pescuma M. Van Nieuwenhove C. Mozzi F. Burgos J. A. S. (2021). Fruits and fruit by-products as sources of bioactive compounds. benefits and trends of lactic acid fermentation in the development of novel fruit-based functional beverages. *Food Res. Int.* 140:109854. 10.1016/j.foodres.2020.109854 33648172

[B39] Shehata M. G. El Sohaimy S. A. El-Sahn M. A. Youssef M. M. (2016). Screening of isolated potential probiotic lactic acid bacteria for cholesterol lowering property and bile salt hydrolase activity. *Ann. Agric. Sci.* 61 65–75.

[B40] Somashekaraiah R. Mottawea W. Gunduraj A. Joshi U. Hammami R. Sreenivasa M. Y. (2021). Probiotic and antifungal attributes of *Levilactobacillus brevis* MYSN105, isolated from an Indian traditional fermented food Pozha. *Front. Microbiol.* 12:696267. 10.3389/fmicb.2021.696267 34290687 PMC8287902

[B41] Somashekaraiah R. Shruthi B. Deepthi B. V. Sreenivasa M. Y. (2019). Probiotic properties of lactic acid bacteria isolated from neera: a naturally fermenting coconut palm nectar. *Front. Microbiol.* 10:1382. 10.3389/fmicb.2019.01382 31316477 PMC6611078

[B42] Steglińska A. Kołtuniak A. Motyl I. Berłowska J. Czyżowska A. Cieciura-Włoch W. (2022). Lactic acid bacteria as biocontrol agents against potato (*Solanum tuberosum* l.) pathogens. *Appl. Sci.* 12:7763.

[B43] Stewart J. E. Thomas K. A. Lawrence C. B. Dang H. Pryor B. M. Timmer L. M. (2013). Signatures of recombination in clonal lineages of the citrus brown spot pathogen, *Alternaria alternata sensu* lato. *Phytopathology* 103 741–749. 10.1094/PHYTO-08-12-0211-R 23441968

[B44] Tanahashi M. Nakano T. Akamatsu H. Kodama M. Otani H. Osaki-Oka K. (2016). *Alternaria alternata* apple pathotype (*A. mali*) causes black spot of European pear. *Eur. J. Plant Pathol.* 145 787–795.

[B45] Taroub B. Salma L. Manel Z. Ouzari H. I. Hamdi Z. Moktar H. (2019). Isolation of lactic acid bacteria from grape fruit: antifungal activities, probiotic properties, and in vitro detoxification of ochratoxin A. *Ann. Microbiol.* 69 17–27.

[B46] Vanitha P. R. Somashekaraiah R. Divyashree S. Pan I. Sreenivasa M. Y. (2023). Antifungal activity of probiotic strain *Lactiplantibacillus plantarum* MYSN7 against *Trichophyton tonsurans*. *Front. Microbiol.* 14:1192449. 10.3389/fmicb.2023.1192449 37389341 PMC10303898

[B47] Viriyasuthee W. Jogloy S. Saksirirat W. Saepaisan S. Gleason M. L. Chen R. S. (2019). Biological control of Alternaria leaf spot caused by *Alternaria* spp. in Jerusalem artichoke (*Helianthus tuberosus* L.) under two fertilization regimes. *Plants* 8:463. 10.3390/plants8110463 31671613 PMC6918389

[B48] Vitali B. Minervini G. Rizzello C. G. Spisni E. Maccaferri S. Brigidi P. (2012). Novel probiotic candidates for humans isolated from raw fruits and vegetables. *Food Microbiol.* 31 116–125. 10.1016/j.fm.2011.12.027 22475949

[B49] Wang H. Yan Y. Wang J. Zhang H. Qi W. (2012). Production and characterization of antifungal compounds produced by *Lactobacillus plantarum* IMAU10014. *PLoS One* 7:e29452. 10.1371/journal.pone.0029452 22276116 PMC3261852

[B50] Wang Y. Wu J. Lv M. Shao Z. Hungwe M. Wang J. (2021). Metabolism characteristics of lactic acid bacteria and the expanding applications in food industry. *Front. Bioeng. Biotechnol.* 9:612285. 10.3389/fbioe.2021.612285 34055755 PMC8149962

[B51] Xu W. Wang H. Lv Z. Shi Y. Wang Z. (2019). Antifungal activity and functional components of cell-free supernatant from *Bacillus amyloliquefaciens* LZN01 inhibit *Fusarium oxysporum* f. sp. niveum growth. *Biotechnol. Biotechnol. Equip.* 33 1042–1052.

[B52] Zhang Y. Yang H. Huang R. Wang X. Ma C. Zhang F. (2022). Effects of *Lactiplantibacillus plantarum* and *Lactiplantibacillus brevis* on fermentation, aerobic stability, and the bacterial community of paper mulberry silage. *Front. Microbiol.* 13:1063914. 10.3389/fmicb.2022.1063914 36483209 PMC9722757

[B53] Zhao H. Liu K. Fan Y. Cao J. Li H. Song W. (2022). Cell-free supernatant of *Bacillus velezensis* suppresses mycelial growth and reduces virulence of *Botrytis cinerea* by inducing oxidative stress. *Front. Microbiol.* 13:980022. 10.3389/fmicb.2022.980022 35992680 PMC9389153

