# Supplementary material for: Antagonistic properties of Lactiplantibacillus plantarum MYSVB1 against Alternaria alternata: a putative probiotic strain isolated from the banyan tree fruit
Source: Front Microbiol. 2024 Feb 9;15:1322758. doi: 10.3389/fmicb.2024.1322758 (PMC10885809; doi:10.3389/fmicb.2024.1322758)
Supplement: Supplementary file 1 [file Data_Sheet_1.docx]

Supplementary Material

Antagonistic properties of *Lactiplantibacillus plantarum* MYSVB1 against *Alternaria alternata*: A putative probiotic strain isolated from the banyan tree fruit

R. Vasundaradevi, M. Sarvajith, R. Somashekaraiah, G. Adithi, M. Y. Sreenivasa*

Applied Mycology Laboratory, Department of Studies in Microbiology, University of Mysore, Manasagangotri, Mysuru-570 006, India

*** Correspondence:**M. Y. Sreenivasa
[sreenivasamy@gmail.com](mailto:sreenivasamy@gmail.com), [mys@microbiology.uni-mysore.ac.in](mailto:mys@microbiology.uni-mysore.ac.in)

Supplementary Table 1. Growth kinetics of *Lpb. plantarum* MYSVA7 and MYSVB1 isolated from Banyan tree fruit.

|  | LAB isolates | |
| --- | --- | --- |
|  | MYSVA7 | MYSVB1 |
| Division rate, R (h^-1^) | 0.83 | 1.05 |
| Specific growth rate, µ (h) | 0.57 | 0.73 |
| Maximum acidification rate* (ΔpH/ Δt) | -0.26 | -0.23 |
| Lowest pH recorded | 3.6±0.1 | 3.8±0.11 |
| Time taken to reach max. acidification (h) | 10 | 10 |

*****Negative values due to decrease in pH.

Supplementary figure 1. Images of the tropical fruits used in this study for the isolation of lactic acid bacteria (LAB). A total of seven different fruits were used (A-G), with the names presented in both common and scientific nomenclature (italics).


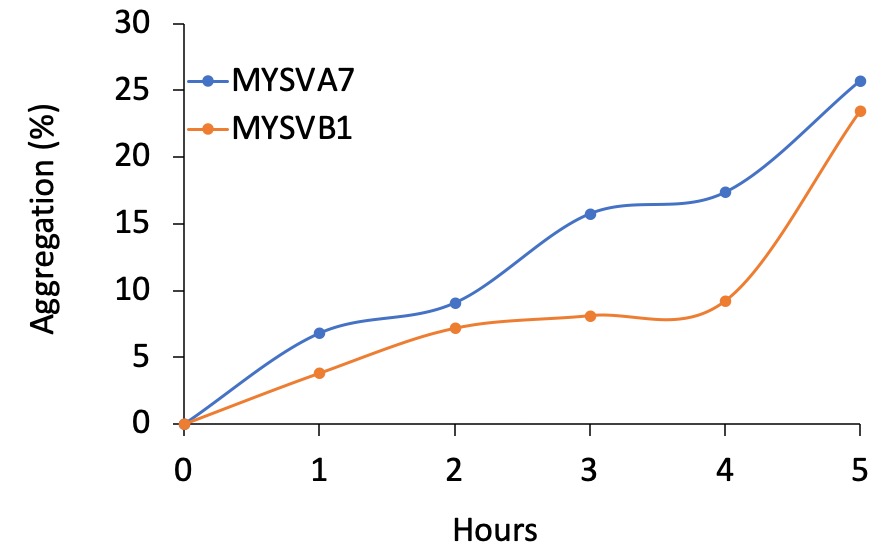


Supplementary figure 2. Time course increase in aggregation ability of the *Lactiplantibacillus plantarum* strains MYSVA7 and MYSVB1.

Supplementary figure 3. Hemolytic activities of isolated *Lactiplantibacillus plantarum* MYSVA7 (A) and MYSVB1 (B) using blood agar media. *Staphylococcus aureus* was used for positive control for (*β*-) haemolysis (C) and an uninoculated plate served as negative control (D).

Supplementary figure 4. Inhibition of growth of *A. alternata* by succinic acid, ASuc (A), lactic acid, ALat (B), and a combination of ASuc and ALac (C).

Supplementary figure 5. Antifungal activity of treated cell-free supernatant (CFS) of *Lactiplantibacillus plantarum* MYSVB1 against *A. alternata*. The pre-treatment of CFS included heat-deactivated CFS (hCFS), proteinase K treatment (pCFS), and pH-neutralization (nCFS).
